# Racialization and Reproduction: Asian Immigrants and California’s Twentieth-Century Eugenic Sterilization Program

**DOI:** 10.1093/sf/soad060

**Published:** 2023-04-29

**Authors:** Marie Kaniecki, Nicole L Novak, Sarah Gao, Natalie Lira, Toni Ann Treviño, Kate O’Connor, Alexandra Minna Stern

**Affiliations:** Institute for Society and Genetics, University of California Los Angeles, Los Angeles, CA 90096, USA; Department of Community and Behavioral Health, College of Public Health, University of Iowa, Iowa City, IA 52242, USA; Center for Population and Development Studies, Harvard T.H. Chan School of Public Health, Harvard University, Boston, MA 02115, USA; Department of Latina/Latino Studies, College of Liberal Arts & Sciences, University of Illinois Urbana-Champaign, Champaign, IL 61820, USA; Department of History, Humanities Programs, Texas Lutheran University, Seguin, TX 78155, USA; Center for Population and Development Studies, Harvard T.H. Chan School of Public Health, Harvard University, Boston, MA 02115, USA; Institute for Society and Genetics, University of California Los Angeles, Los Angeles, CA 90096, USA

## Abstract

During the twentieth century, state health authorities in California recommended sterilization for over 20,000 individuals held in state institutions. Asian immigrants occupied a marginalized position in racial, gender, and class hierarchies in California at the height of its eugenic sterilization program. Scholars have documented the disproportionate sterilization of other racialized groups, but little research exists connecting the racist, gendered implementation of Asian immigration restriction to the racism and sexism inherent in eugenics. This study examines patterns of coercive sterilization in Asian immigrants in California, hypothesizing higher institutionalization and sterilization rates among Asian-born compared with other foreign- and US-born individuals. We used complete count census microdata from 1910 to 1940 and digitized sterilization recommendation forms from 1920 to 1945 to model relative institutionalization and sterilization rates of Asian-born, other foreign-born, and US-born populations, stratified by gender. Other foreign-born men and women had the highest institutionalization rates in all four census years. Sterilization rates were higher for Asian-born women compared with US-born [Incidence Rate Ratio (IRR) = 2.00 (95% CI: 1.61, 2.48)] and other foreign-born women (*p* < 0.001) across the entire study period. Sterilization rates for Asian-born men were not significantly higher than those of US-born men [IRR 0.95 (95% CI 0.83, 1.10). However, an inflection point model incorporating the year of sterilization found higher sterilization rates for Asian-born men than for US-born men prior to 1933 [IRR 1.31 (95% CI 1.09, 1.59)]. This original quantitative analysis contributes to the literature demonstrating the health impact of discrimination on Asian-Americans and the disproportionate sterilization of racial minorities under state eugenics programs.

## Introduction

The COVID-19 pandemic prompted increased xenophobia and racialized violence toward Asian-Americans and Asian immigrants in the United States. This surge in discrimination and hate crimes is unsurprising when tracing long-standing stereotypes of Asians as threats to public health. Historians have documented the development of legal, social, and scientific narratives portraying Asians, both foreign- and American-born, as unassimilable aliens and threats to the health of white populations (Ngai 2014; Stern 2016). Public health policies and practices reified the racialization of Asians through “yellow peril” narratives, cleanliness crusades, and birth rate monitoring (Molina 2006; Ngai 2014; Shah 2001; Takaki 1998). Despite this historical literature, many discussions about systemic racism and discrimination in health in the United States overlook Asians, reflected in limited public health literature about this topic (Gee et al. 2009). Our research addresses this gap by analyzing the eugenic sterilization and institutionalization of Asian immigrants in twentieth-century California. While public health and medicine no longer explicitly espouse eugenic theories, systemic biases against poor people, people of color, and disabled people remain inherent in many public health systems, such as COVID-19 triage policies prioritizing non-disabled people in the rationing of care. Studying the influence of eugenics on public health practice—as in state-mandated sterilization programs—can produce a greater understanding of continued discrimination against Asians in the United States and potentially inform efforts to combat such inequities in public health.

This is the first study to quantitatively analyze the eugenic sterilization of Asian immigrants in the twentieth century. The impact of California’s twentieth-century eugenic sterilization program on Asians merits closer examination given the racist implementation of eugenic programs and the sociopolitical position of Asians in California’s racial, gender, and class hierarchies at the time. Previous studies reported a disproportionate sterilization of Black people in North Carolina (Price and Darity 2010; Price, Darity, and Sharpe 2020) and Spanish-surname people in California (Novak et al. 2018) under each state’s respective eugenic sterilization program, exemplifying how policies codifying categories of disability, without explicit mentions of race or ethnicity, could none the less result in medical racism (Lira 2022). The historical context and documented disproportionate sterilization of other minority groups prompts the following research questions: did Asian immigrants have disproportionate institutionalization rates in California, and did rates vary among Asian national origin groups? Did Asian immigrants have disproportionate sterilization rates in California institutions? Did demographic and social characteristics differ for Asian-born versus other foreign- and US-born patients recommended for sterilization?

### Racial Formation and “Asian Nativity”

Our study analyzes “Asian race” as a socially and historically constructed category rather than a biological reality (Omi and Winant 2015). Although not biological, racial categories like “Asian” merit careful inclusion in retrospective studies like ours to better understand racism in public health and medicine (Roberts 2011). Historians have documented the socio-legal formation of the concept of “Asian race” in the United States during the nineteenth and twentieth centuries whereby people with varied national and ethnic backgrounds came to constitute a racialized group (Lee 2010; Ngai 2014; Shah 2001; Takaki 1998). As a racialized group, Asian immigrants and their American-born children on the West Coast disrupted the predominant American narrative of the black/white racial dichotomy (Molina 2006). Ultimately, “Asian” as a racial category was incorporated into and thus reaffirmed the existence of a racial hierarchy (Espiritu 1997).

The racial formation of “Asian” immigrants occurred through strict federal and state legislation over six decades that excluded and grouped people from different countries of origin—primarily people from China, India, Japan, Korea, and the Philippines (Molina 2006; Ngai 2014; Shah 2001; Takaki 1998). Exclusionary policies included the 1882 Chinese Exclusion Act (Ngai 2014; Shah 2001), the 1908 Gentlemen’s Agreement limiting Japanese immigration (Molina 2006; Ngai 2014), the 1917 Asiatic Barred Zone tying exclusion to geography (Bennett 2017), and several Alien Land Laws banning Asian immigrants from owning land. Nativist organizations such as the Asiatic Exclusion League advocated for segregation of Asian children in public schools and fueled anti-Asian sentiment among the public (Shah 2001; Takaki 1998). In addition to restrictive immigration legislation, state and federal courts and public institutions helped construct and reify “Asian” as a racial category. Judges employed a flexible “common knowledge” delineation of race in several court cases to deny citizenship to Easurk Emsen Charr, a Korean-born World War I US military veteran, Takao Ozawa, a Japanese man (*Ozawa v. United States* 1922), and Bhagat Singh Thind, an Indian man (United States v. Thind 1923) (Ngai 2014; Takaki 1998). These cases established a legal precedent designating all Asian immigrants as neither black, nor white and thus racially ineligible for US citizenship (Molina 2006). By the time of the Immigration Act of 1924, designed in part by advocates of eugenics, US courts determined that immigrants from various countries designated “Asian” or “Oriental” were racially distinct, inherently foreign, permanently unassimilable, and undesirable (Ngai 2014).

This condensed history demonstrates the formation of the socially constructed racial category of “Asian” during the eugenics era. Given this history, our paper uses nativity from China, Japan, Korea, India, and the Philippines—what we label “Asian-born”—as a proxy for the way these individuals were racialized by institutional authorities making decisions about whether they should be sterilized. This category derives meaning and power not from the shared continent of Asian immigrants’ countries of origins but from the shared experiences of Asian immigrants with discrimination and racialization in American society (Chan 2007). Although other nativity groups were targeted for exclusion under various anti-Asian legislations, they did not have as prominent a history of exclusion or the same level of examination in the literature. Though the racialization of these Asian subgroups converged during the study period in service of exclusion, the boundaries of the “Asian race” continued to fluctuate. Ethnic differentiation among these subgroups may have subsumed the prominence of the racialized group in certain times and contexts (Lee 2010). Although we examine their experiences as a group, we also disaggregate by subgroup when the data allow (Chan 2007). The following subsections will expand on the role of eugenicists in constructing immigrants labeled “Asian” as biologically distinct and genetically inferior.

### Institutionalization and Eugenic Sterilization

Proponents of eugenics blamed “bad genetics” for social issues, including criminality (Gault and Laughlin 1924; Stern et al. 2017), sexual deviancy (Gault and Laughlin 1924; Kline 2001; Stern 2005), and poverty (Gault and Laughlin 1924; Price and Darity 2010). Eugenicists advocated for a wide array of eugenic measures, encouraging those considered “genetically fit” to reproduce more and using policies of social exclusion and reproductive control to prevent people considered “genetically unfit” from having children (Stern 2016). Institutionalization served as one such form of eugenic population control, whereby people deemed “unfit” were segregated from the general population, separated by sex, and banned from engaging in sexual activity (Radford 1991).

The varied pathways into state institutions included commitment by a family member such as a spouse or parent, forced placement by a juvenile official usually involving court proceedings, or transfer, particularly of young people, from a reform school (Chavez-Garcia 2012; Lira 2022). Less commonly, individuals committed themselves to institutions. Once in an institution, an individual was effectively incarcerated and unable to leave until discharged by authorities. “Feeblemindedness” was the most pervasive diagnostic label used by medical professionals, describing a patient’s perceived inability to engage in productive work or conform to normative behaviors (Kline 2001). Typically derived by scoring a mental test to rank IQ, the broad category of “feebleminded” encompassed subcategories such as moron, imbecile, or idiot, all psychometric labels for IQs in the 20–80 range below the “normal” score of 100. Those committed to insane hospitals were classified as suffering from psychiatric conditions including dementia praecox (a precursor term for schizophrenia) and psychosis. Historians have shown these diagnoses were extensively applied to marginalized groups including people with disabilities, the indigent, gender non-conforming people, and people deemed socially or morally deviant (Lira 2022; Trent 1995). Eugenicists theorized that these conditions were hereditary and used “feeblemindedness” and mental deficiency as flexible concepts to mark a wide range of individuals for institutionalization and eugenic sterilization.

The advent of sterilization fundamentally shifted the institutionalization process and offered institutional authorities a mechanism to “safely” release some patients without fear they would reproduce (Radford 1991; Reilly 2015). Thirty-two US states enacted eugenic sterilization laws in the 1900s, resulting in an estimated 60,000 coercive sterilizations officially reported nationwide; California performed one-third of these procedures (Stern 2016). Individuals sterilized by state-mandated sterilization were frequently labeled “dangerous to public health,” explicitly invoking public health as grounds for violating their reproductive rights (Stern et al. 2017, 50).

Institutionalization in state hospitals for the insane or homes for the “feebleminded” constituted the primary setting for eugenic sterilization in California (Stern 2005). In one Department of Institutions report, institutional authorities described the procedure as an important tool for preventing “the propagation of the mentally and physically unfit” (California State Printing Office 1926, 96). Many institutions implemented this tool by performing sterilizations prior to release (Lira 2022; Stern 2011). For example, Norwalk State Hospital’s medical superintendent asserted “every person of the proper age should be sterilized before leaving an institution of this kind” (California State Printing Office 1924, 89). Describing Sonoma State Home’s standard discharge procedures since 1918, Dr. Fred O. Butler stated the “majority” of patients were “sterilized before being paroled,” (California State Printing Office 1924, 94). Pacific Colony, California’s other home for the feebleminded, had a similar de facto policy to sterilize patients prior to release. Researchers of other California State institutions observed sterilization “routinely performed as a precondition to discharge” (Edgerton 1993, 103; Rouble 1942, 3 and 55). Thus, people committed to California state institutions were at risk of being sterilized and sterilizations were largely performed on patients to enable discharge.

California’s eugenic sterilization law granted institutional superintendents’ discretion to authorize the sterilization of any individual committed to a state institution. Given this intersection of institutionalization and sterilization in California, we analyze rates of both. We use sterilization request forms (also known as recommendation forms) created by institutional and state authorities for institutionalized individuals deemed “unfit” to reproduce. See the online supplementary material for Figure S1. Although not proof of sterilization, the forms were the main mechanism through which compulsory sterilizations were authorized in California. Moreover, the forms contain demographic and diagnostic information documented by psychiatrists and clinicians, and social information gathered from social workers and other state authorities.

### Race and Immigration Status as Exposures During the Eugenics Movement

Disability diagnoses and labels popular in the early twentieth century—like “feeblemindedness” or “dementia praecox”—often led to institutionalization. Racism, classism, sexism, and xenophobia not only shaped understandings of who was considered “unfit” but also the development and application of disability labels indicating mental illness or cognitive impairment (Baynton 2016; Dorr 2008; Kline 2001; Lira 2022; Metzl 2011; Rembis 2013; Trent 1995). Eugenicists constructed a fitness hierarchy whereby Anglo-American, white, upper- or middle-class, able-bodied, and neurotypical people were idealized and encouraged to reproduce. Immigrants and other racialized groups within the United States were situated at the bottom of this hierarchy, not only cast as genetically inferior but also accused of being more prone to “feeblemindedness” and mental illness, thus justifying eugenic policies like immigration restriction, institutionalization, and sterilization (Novak et al. 2018; Price and Darity 2010; Price, Darity Jr., and Sharpe 2020). In this paper, we analyze Asians as a significant racialized immigrant group exposed to eugenic policies in California. Our analysis builds on extant historical literature on the intersections of race, immigration, disability, and public health.

Disparaging associations of Asian race and disability developed before the height of the eugenics movement. Prior to the discovery that trisomy 21 caused the condition known as Down’s Syndrome, Dr. John Langdon Down theorized racial degeneration caused this condition to develop among Europeans. He compared the physical features of people with trisomy 21 to the physcial features of people from Mongolia and asserted “a very large number of congenital idiots are typical Mongols” (Down 1866, 2). “Mongolian” was also inconsistently used to describe other Asian ethnic groups in nineteenth- and twentieth-century racial lexicons, especially Chinese people. In the late nineteenth century, American physicians adopted the terms Mongol, Mongoloid, and Mongolism to label people with trisomy 21; their use persisted into the 1900s even after the discovery of Down syndrome’s genetic origin. During the eugenics era, “mongolism” was discussed as a proof of hereditary taint (Wright 2004).

Public health rhetoric expanded xenophobic “Yellow Peril” narratives, which positioned Asian immigration as threatening the US social order, into the realm of medicine. Nativists and eugenicists alike cast racialized Asian immigrants as inferior and undesirable by linking them to disability and illness. Officials believed Asians were hereditarily prone to diseases such as trachoma and syphilis and would eventually spread them to white populations (Shah 2001). Increased control of the nation’s borders to prevent inflows of allegedly diseased immigrants thus became integral to the project to protect public health, resulting in the medicalization of US international borders (Baynton 2016; Fairchild 2003; Markel and Stern 1999; Mckiernan-González 2012). Beginning in the early 1900s, Angel Island in San Francisco Bay functioned as a public health quarantine station and a major exclusion and detention site for Asian immigrants (Gee 1999; Markel and Stern 1999; Shah 2001). Public Health inspectors excluded immigrants seeking admittance if invasive medical exams found them “likely to be public charges” because of assumed disability or chronic disease.

California institutional officials often blamed foreign-born populations for California’s supposed high insanity rates (Appleman 2018; Fox 1978; Simon and Rosenbaum 2012). During the eugenics era, the California Department of Institutions established the Office of the Deportation Agent within state institutions (Stern 2016), signifying heightened concern about immigrant mental illness and disability. Thus, institutionalization and sterilization operated alongside broader efforts to manage and exclude immigrants deemed racially “unfit” (Toner and Norman 1932). Given these racist and xenophobic associations of Asian immigrants with disease and disability and the centrality of disability in eugenic practices, Asians were likely targets of institutionalization and sterilization in California.

### Sex and Gender in Sterilization and the Racialization of Asian Immigrants

Race and gender are not mutually exclusive categories but instead exist as overlapping “structures of difference and disempowerment [that] reinforce one another” (Espiritu 1997, 15). The intersectionality theory (Crenshaw 2018) and social dominance theory (Sidanius et al. 2018) both assert that gender strongly influences racialization. The subordinate male target hypothesis, derived from the social dominance theory, posits the men of a racially defined outgroup would be primary targets of intergroup discrimination informed by perceived sexual and reproductive threat posed to the ingroup (Navarrete et al. 2010; Sidanius et al. 2018). Liu and Wong’s (2018) review of empirical studies found that Intersectional Fusion Paradigms better explained Asian-American men’s experiences with discrimination than the subordinate male target hypothesis. One such paradigm is the Gendered Racism Model, whereby the social meanings of race and gender define and reinforce each other, cannot be fully disentangled, and create unique systems of discrimination for individuals with different combinations of social identities. Racism faced by Asian immigrant men and women could therefore manifest in qualitatively, rather than quantitatively, different ways (Liu and Wong 2018). Under each of these theories, Asian Americans occupy space as multiply disadvantaged people. Thus, a failure to consider the intersections of gender and race further marginalizes the experiences of Asian immigrants (Espiritu 1997).

The literature on both eugenic sterilization (Kline 2001; Lira 2022; Stern 2011; Stern 2016) and Asian immigration (Espiritu 1997; J. Gee 2003; Lee 2010; Lee 2003a; E. Lee 2003b; Liu and Wong 2018; Mabalon 2013; Shah 2001) to the United States indicates a need for gendered analysis. In both cases, ostensibly gender-neutral legislation resulted in gendered implementation. Differences in surgical procedure for vasectomies, hysterectomies, and tubal ligations and strict socially prescribed gender roles led to a distinctively and consciously gendered enactment of sterilization programs (Stern 2016). For example, Kline (2001) describes how the Sonoma State Home for the Feebleminded centered sexual promiscuity and deviation from domestic norms to justify sterilizing women. In contrast, men were often admitted to Sonoma for criminal behavior but were ultimately sterilized for supposedly “therapeutic” reasons. Developments in surgical technique made female sterilizations easier, enabling an increased focus on women in later years of eugenic sterilization programs (Stern 2016). In this paper, sex and gender are used jointly and interchangeably, acknowledging overlapping biological and social aspects of sterilization and a synonymous use of the terms during the study period.

Men vastly outnumbered women among Asian immigrants in the United States, though the sex ratio became less skewed over time (Cheng and Bonacich 1984; Lee 2010; Lee 2003a, Lee 2015; Takaki 1998; Shah 2001). In addition to gendered logics operating within the US immigration system, gendered dynamics in immigrant countries of origin influenced immigrant selectivity prior to arrival (Lee 2010). Asian men typically emigrated as wage-earning sojourners, whereas maintaining respectability required Asian women to remain in the family home instead of traveling abroad (Lee 2010; Lee 2003a, Lee 2003b, Lee 2015). In contrast to typical characterizations of Asian immigrant men as mostly bachelors, many left behind wives and children in their countries of origin, leading to widespread and often long-term family separation (Lee 2010; E. Lee 2003b). Surprisingly, Angel Island interrogation transcripts of Asian men revealed little concern over their sexuality from immigration officials, who instead focused on determining the socioeconomic status and enforcing exclusion through the “public charge” provision (Gee 2003). Nevertheless, once past ports of entry, the disproportionately male Asian immigrant population fed distinct concerns about deviant sexuality and race mixing directed toward Asian men in American society. Asian men were stereotyped as feminized and androgynous (Ngai 2014), deviating from traditional masculinity and invoking fears of homosexuality (Shah 2001; Shah 2005). California’s nineteenth century anti-miscegenation laws forbid marriage between whites and “Mongolians,” and Filipino men were especially characterized as threats to white women (Ngai 2014; Sohoni 2007). The 1922 Cable Act further deterred interracial relationships between US-born women of many races and Asian-born men—women who married men racially ineligible to citizenship (i.e. Asian men) lost their own US citizenship (Lee 2003b).

Many Asian women immigrated to the United States during the era of exclusion through exemption clauses allowing immigrant (pre-exclusion or exempt class) or US-born Asian men to bring over wives, children, or other family members (Gee 2003, 1999; Lee 2003a, Lee 2003b). However, the everyday decision-making of immigration officials handling their cases ultimately determined whether Asian women were detained or allowed to enter (Lee 2003b). These officials deftly wielded an array of laws in “constricting the loophole for exempted wives” in practice (Gee 2003, 97). Asian women’s entry to the United States often rested upon their ability to perform respectable femininity during interactions with the immigration enforcement system (Gee 2003; Lee 2003b). The 1875 Page Act was the first legislation to use narratives surrounding Asian women and deviant sexuality to allow immigration officials to deny immigrants entry, impose prolonged detention, or subject women to invasive questioning and medical examinations (Lee 2003a; Shah 2001). These conditions deterred many Asian women from immigrating (Lee 2003a). Angel Island is typically foregrounded as a site of Chinese exclusion, but gendered gatekeeping processes operated there for Asian women of many national origins (Gee 1999, 2003; Lee 2015; Mabalon 2013; Shah 2001). Gendered logics initially supported ethnic differentiation between Chinese and Japanese women in the immigration process (Lee 2010). Public health discourse condemned Chinese women as “dangerous” and “syphilitic” prostitutes (Lee 2010; Ngai 2014; Shah 2001). In contrast, during interrogations, Japanese women often discussed their intentions to become housewives after admittance, which immigration officials believed to be respectable domestic labor and indicative of both gender and class status (Gee 2003). With increased Japanese settlement and the participation of Japanese women in (traditionally masculine) agricultural labor on family land (Gee 1999; Molina 2006), this positive view of Japanese women eventually disappeared (Lee 2010). This converging racialization of earlier Asian immigrant women from different national backgrounds created a framework through which to incorporate additional ethnic groups into existing prejudices and exclusionary practices (Lee 2010), constructing Asian women as “racially prone to immorality” (Gee 2003, 102), and therefore, undesirable.

California officials were also preoccupied with the sexual and reproductive lives of Asian immigrant families. The gender imbalance among Asian immigrants led to widespread non-normative family structures that deviated from the “Western” nuclear ideal. The supposed prevalence of prostitutes and “concubines” in Chinese communities implied that neither Chinese women nor men respected the sanctity of marriage (Lee 2010). Some Chinese women and children resided in multi-family households, occasionally without a male head of household, enacting what Shah labels “queer domesticity” (2001). Filipino and South Asian families created extensive kinship networks of godparents or “uncles” of non-blood-related men in the community (Lee 2015; Mabalon 2013; Shah 2011). Over time, the Asian-American population had shifted from “single” male laborers to family units (Lee 2010; Lee 2003a, 2003b; Shah 2011), signifying permanent settlement and enabling the birth of a second generation of US citizens. Japanese immigrant women, disparaged for neglecting domestic duties through participation in agricultural labor (Gee 1999; Molina 2006), also prompted the suspicion of an intentional colonization of the United States through “explosive” population growth (Gee 2003; Lee 2010). Los Angeles public health officials feared purportedly high Japanese birth rates and reported such statistics in formats exaggerating population growth (Molina 2006). Previously, the scarcity of family units provided “evidence” that Asian immigrants were unassimilable into American society (Molina 2006; Ngai 2014; Shah 2001), but anti-Asian rhetoric adapted to the demographic transition. Family formation and reproduction were closely tied to national identity—non-white immigrant families endangered the white national identity of the United States by threatening to outpace white population growth and reducing land available for the settlement of white families (Lee 2010). American society feared not only the existence of Asian-American families but also the creation of families with large numbers of non-white children (Lee 2010). The California Alien Land Laws prohibited the ownership or long-term leases of land by those racially ineligible to citizenship (i.e. Asian immigrants) to preserve white land ownership. US citizen children became the primary mechanism for Asian immigrants to circumvent the land laws (Gee 1999; Lee 2010; Lee 2010; Mabalon 2013; Molina 2006; Ngai 2014; Pido 2016; Shah 2011; Takaki 1998), inadvertently linking Asian land ownership to Asian reproduction (Lee 2010).

### Hypothesized Patterns of Eugenic Sterilization among Asian Immigrants in California

Since eugenic definitions of “unfit” overlapped with racialized characteristics ascribed to Asian immigrants, we theorize Asian-born people had overall higher rates of institutionalization and eugenic sterilization from 1920 to 1945 compared with non-Asian-born people (other foreign- and US-born), with gender modifying the relationship; however, we postulate heterogeneity in institutionalization rates in Asian-born individuals by gender and nativity subgroup. Initial analysis prompted an additional post hoc hypothesis that the year of sterilization recommendation could modify the relationship between nativity and the sterilization rate for men.

## Methods

### Data

This retrospective population study used two datasets: complete-count US decennial census microdata (Ruggles et al. 2021) from 1910 to 1940 and a dataset constructed from over 20,000 digitized sterilization recommendation forms from the California Department of Institutions (now the Department of State Hospitals) using REDCap software (Harris et al. 2009). Census microdata were used to calculate institutionalization rates and provide denominators for sterilization rates since individual-level institutional records were not available for the entire institutionalized population (i.e. the full population at risk of sterilization).

Sterilization requests are assumed to be evidence of sterilization as an integral step in the state-sponsored sterilization process in California. These recommendations were the primary mechanism through which compulsory sterilizations were authorized in institutions, and all included requests were approved by the Department of Institutions. These forms contained demographic, personal, and familial information recorded by institutions. Sterilization records in the dataset were centralized from eleven California institutions during the 1920s transition from the California Commission in Lunacy to the California Department of Institutions. Two institutions were Homes for the Feebleminded (Sonoma and Pacific Colony), and nine were State Hospitals for the Insane (Patton, Stockton, Norwalk, Agnews, Mendocino, Camarillo, Napa, Dewitt, and Modesto). Dewitt and Modesto State Hospitals did not recommend any sterilizations until after 1945 and are therefore not included in the analysis.

For more details about data collection and digitization, see the methodological supplement of Stern et al. (2017). The digitization process followed institutional review board protocols of the University of Michigan and the California Committee for the Protection of Human Subjects. All statistical analyses were performed using Stata version 16 (StataCorp LP, College Station, TX).

### Independent Variable: Asian Nativity

In both datasets, we used data on birthplace to generate a three-category nativity variable: (i) US-born; (ii) Chinese-, Indian-, Japanese-, Korean-, or Filipino-born; or (iii) other foreign-born. US-born included people born in US territories granting birthright citizenship during the study period (e.g. Hawaii) but not US territories whose residents were excluded from citizenship (e.g. the Philippines).

### Descriptive Statistics: Census Data

We restricted the census data to those with complete data on sex and birthplace.

Bivariate statistics were calculated for age, census year, institution, and sex by the nativity group for the institutionalized population (*n*_1920_ = 11,396, *n*_1930_ = 16,801, *n*_1940_ = 26,748) and overall population of California (*n*_1910_ = 2,380,063, *n*_1920_ = 3,433,668, *n*_1930_ = 5,669,757, *n*_1940_ = 6,879,664).

### Analysis: Institutionalization Rates

To estimate institutionalization rates across nativity groups, we collapsed census microdata to generate counts of California’s institutionalized population and overall population, by gender and nativity group, for each census year. We used Poisson regression to compare institutionalization rates between nativity groups in each census year, using the count of institutionalized persons as the dependent variable, the count of total California residents as the offset, and the nativity group as the independent variable. For regression coefficients estimating differences in institutionalization rates by nativity, US-born was the reference group. A post hoc test evaluated whether differences in regression coefficients for Asian- and other foreign-born people were statistically significant. We conducted additional analyses disaggregating the Asian-born group into Chinese-, Indian-, Japanese-, Korean-, or Filipino-born to interrogate nationality differences in the institutionalization of this racialized group. For all models, we exponentiated regression coefficients to calculate incidence rate ratios comparing institutionalization rates for Asian- and other foreign-born to those of US-born persons.

### Descriptive Statistics: Sterilization Data

The sterilization dataset was restricted to 17,813 people recommended for sterilization from 1920 to 1945 corresponding with available US Census microdata from 1920, 1930, and 1940. Those missing sex (*n* = 32) and nativity (*n* = 4282) information were excluded. Since sterilization recommendation forms did not systematically report race or ethnicity, we designated Chinese-, Indian-, Japanese-, Korean-, or Filipino-born individuals as part of a racialized “Asian-born” group (*n* = 287). A categorical nativity variable identified Asian-born people, other foreign- (*n* = 2303), and US-born people (*n* = 10,909). The final analytic sample was *n* = 13,499.

Descriptive statistics were calculated among the population recommended for sterilization, stratified by the nativity group, for basic demographic information (age, period, institution, and sex), patient histories, diagnosis, legal provision, and consent. A Holm–Bonferroni correction for multiple testing was applied to all statistical tests of descriptives besides basic demographics. Additional methodological information for descriptive statistics can be found online.

### Analysis: Sterilization Rates

To estimate sterilization rates across nativity groups, we collapsed our microdata to generate counts of sterilization recommendations, by gender, nativity group, and year from 1920 to 1945. We similarly collapsed census microdata to generate counts of institutionalized persons in 1920, 1930, and 1940 by sex and nativity group. To generate intercensal year counts, we linearly interpolated the number of institutionalized people, by gender and nativity group, between census years. Counts from 1940 were extended forward to 1945. For hospitals established after 1920 (Pacific Colony in 1927 and Camarillo in 1933), institutionalized populations were interpolated back from the first census after opening.

Because sterilization practices differed between males and females, we stratified all analyses on sex. To qualitatively demonstrate time trends, locally estimated scatterplot smoothing (LOESS) curves were constructed for annual population-based sterilization rates, stratified by nativity group and sex. We used Poisson regression to compare sterilization rates between nativity groups, using the annual number of sterilizations as the dependent variable, the number of institutionalized persons as the offset, and the nativity group as the independent variable. For regression coefficients estimating differences in sterilization rates by nativity, US-born was the reference group. A post hoc test evaluated whether regression coefficients for Asian- and other foreign-born people differed statistically. LOESS curves and regression models could not be adjusted for age or institution or disaggregated by the Asian nativity subgroup, as small cell sizes prevented the reliable interpolation of census denominators. A post hoc analysis for the effect modification of the sterilization rate model by time for men only in the form of a 1933 inflection point was conducted based on observations of LOESS curves.

A sensitivity analysis was conducted to examine the potential impact of missing data on the results. Additional Poisson regressions modeled low and high estimates of the number of Asian-born patients among individuals missing nativity information (*n* = 4,282). The online supplement details our methods for generating lower and upper bound estimates.

## Results

### Descriptive Statistics: Census Data

Table 1 displays demographic characteristics and institutionalization status, by the nativity group, in California’s overall population in census years from 1910 to 1940 (*n*_1910_ = 2,379,316, *n*_1920_ = 3,408,544, *n*_1930_ = 5,669,480, *n*_1940_ = 6,879,092). Across the four censuses, California’s Asian-born population was 79.14% male, whereas other foreign-born people were 57.26% and US-born people were 50.46% male. California’s US-born population increased each census year from 1910 to 1940, and its Asian-and other foreign-born population decreased from 1930 to 1940. The proportion of the population institutionalized across the four census years varied by nativity group, with the greatest proportion institutionalized among other foreign-born, followed by Asian- and then US-born persons.

**Table 1:** Sex and Year Distribution of California’s Overall Population by Nativity for the 1910 (*n* = 2,379,316), 1920 (*n* = 3,408,544), 1930 (*n* = 5,669,480), and 1940 (*n* = 6,879,092) censuses (Total *n* = 18,336,432)

	Asian nativity	Other foreign nativity	US nativity	
	*N* (%)	*N* (%)	*N* (%)	*Χ* ^2^ *P*-value
Sex				
Female	69,923 (20.86%)	1,334,408 (42.74%)	7,370,836 (49.54%)	*p* < 0.001
Male	265,294 (79.14%)	1,788,025 (57.26%)	7,507,946 (50.46%)	
Year				
1910	68,724 (20.50%)	527,084 (16.88%)	1,783,508 (11.99%)	*p* < 0.001
1920	77,010 (22.97%)	694,486 (22.24%)	2,637,048 (17.72%)	
1930	102,584 (30.60%)	1,016,089 (32.54%)	4,550,807 (30.59%)	
1940	86,899 (25.92%)	884,774 (28.34%)	5,907,419 (39.70%)	
Institutionalization status				
Institutionalized	1,177 (0.36%)	18,288 (0.59%)	42,995 (0.29%)	*p* < 0.001
Non-institutionalized	334,040 (99.65%)	3,104,145 (99.41%)	14,835,787 (99.71%)	
TOTAL	335,217	3,122,433	14,878,782	—

Table 2 displays demographic characteristics of California’s institutionalized population across all censuses (*n*_1920_ = 11,396, *n*_1930_ = 16,801, *n*_1940_ = 26,748) by nativity group (*n*_Asian nativity_ = 891, *n*_other foreign-born_ = 15,242, *n*_US-born_ = 38,812) and chi-squared *P*-values. There were statistically significant differences in age distribution by nativity, with Asian-born institutionalized persons tending to be older than US-born but younger than other foreign-born people. A greater proportion of Asian-born patients were men (79.96%) than among other foreign-born (60.77%) or US-born (50.51%) people. The institutions housing the most Asian-born patients during census years were Stockton State Hospital (29.85% of Asian-born patients), Napa State Hospital (14.93%), Patton State Hospital (16.50%), and Mendocino State Hospital (15.15%). A greater proportion of Asian-born patients were institutionalized at Stockton than were other foreign- (21.66%) or US-born (13.13%) persons. Asian-born patients were primarily institutionalized in “Hospitals for the Insane” with only 2.00% of Asian-born patients at Sonoma and Pacific Colony. This proportion is lower than the 20.12% of US-born patients in Homes for the Feebleminded. The institutionalized population increased each census for all nativity groups.

**Table 2:** Demographic Characteristics of California’s Institutionalized Population by Nativity for the 1920 (*n* = 11,396), 1930 (*n* = 16,801), and 1940 (*n* = 26,748) censuses (Total *n* = 54,945)

	Asian-born	Other foreign-born	US-born	
	*N* (%)	*N* (%)	*N* (%)	*Χ* ^2^ *P*-value
Age				
<20	11 (1.28%)	141 (0.94%)	3,684 (9.84%)	—
20–29	100 (11.66%)	803 (5.33%)	5,342 (14.28%)	—
30–39	229 (26.69%)	2,406 (15.96%)	7,466 (19.95%)	*p* < 0.001
40–49	219 (25.52%)	3,660 (24.29%)	7,828 (20.92%)	—
50–59	163 (19.00%)	3,811 (25.29%)	6,663 (17.81%)	—
60–69	99 (11.54%)	2,657 (17.63%)	4,190 (11.20%)	—
70+	37 (4.31%)	1,593 (10.57%)	2,248 (6.01%)	—
Total	858	15,071	37,421	—
Sex				
Female	137 (15.38%)	5,979 (39.23%)	19,193 (49.45%)	*p* < 0.001
Male	754 (84.62%)	9,263 (60.77%)	19,619 (50.55%)	—
Total	891	15,242	38,812	—
Institution				
Homes for the Feebleminded				
Sonoma	17 (1.91%)	350 (2.30%)	6,382 (16.47%)	—
Pacific Colony	1 (0.11%)	65 (0.43%)	1,427 (3.68%)	*p* < 0.001
Total, Homes for the Feebleminded	18 (2.00%)	415 (2.73%)	7,809 (20.12%)	—
Hospitals for the Insane				
Patton	147 (16.50%)	2,605 (17.12%)	6,497 (16.76%)	—
Stockton	266 (29.85%)	3,296 (21.66%)	5,087 (13.13%)	—
Norwalk	63 (7.07%)	1,257 (8.26%)	3,458 (8.92%)	—
Agnews	92 (10.33%)	1,967 (12.93%)	5,169 (13.34%)	—
Mendocino	135 (15.15%)	2356 (15.48%)	2,928 (7.55%)	—
Camarillo	37 (4.15%)	624 (4.10%)	1,923 (4.96%)	—
Napa	133 (14.93%)	2,698 (17.73%)	5,885 (15.18%)	—
Total, Hospitals for the Insane	873 (98.00%)	14,803 (97.27%)	31,003 (79.88%)	—
Total, all institutionalized	891	15,242	38,812	—
Year				
1920	181 (20.31%)	4,092 (26.85%)	7,123 (18.35%)	—
1930	282 (31.65%)	4,373 (28.69%)	12,146 (31.29%)	*p* < 0.001
1940	428 (48.04%)	6,777 (44.46%)	19,543 (50.35%)	—
Total	891	15,242	38,812	—

### Institutionalization Rate Analysis

**Figure 1:**
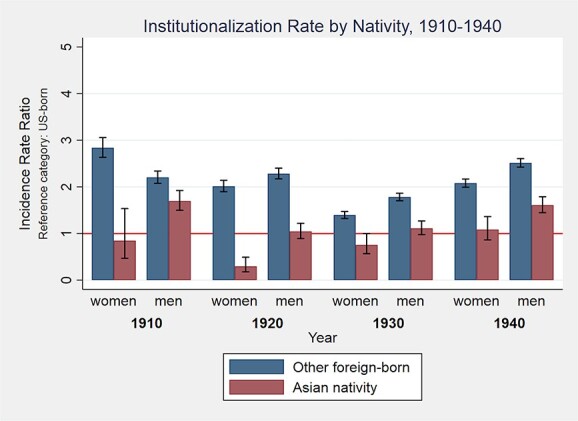
Institutionalization rates by nativity group and sex, 1910–1940.

Figure 1 presents results of Poisson regressions comparing institutionalization rates for each census year, stratified by sex and nativity group, with US-born as the reference category. Other foreign-born men and women had higher institutionalization rates than their US- and Asian-born counterparts in all four census years. Asian- and US-born women had statistically similar institutionalization rates every census year except 1920, when Asian-born women had a lower rate. Asian-born men had significantly higher institutionalization rates in 1910 and 1940 and statistically similar rates in 1920 and 1930 relative to US-born men.

**Figure 2:**
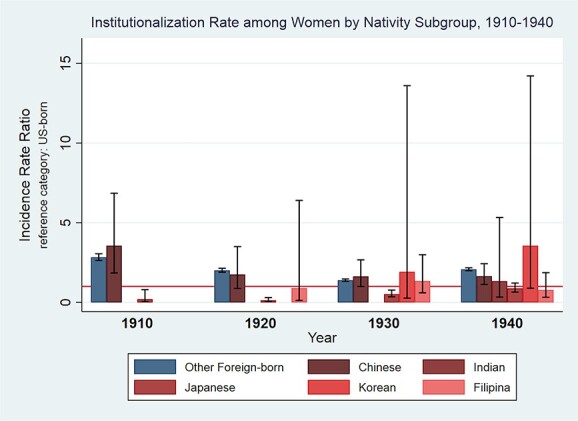
Institutionalization rates by nativity subgroup, women, 1910–1940.

Figure 2 displays results of Poisson regressions disaggregating the Asian nativity group and comparing institutionalization rates for each subgroup to US-born women. The census did not report any Filipina women institutionalized in 1910, Korean women institutionalized in 1910–1920, or Indian women institutionalized in 1910–1930. In 1910, Japanese-born women had the lowest institutionalization rate. Japanese women’s institutionalization rate remained low in 1920 but increased to be comparable to other groups by 1940. Among women, no Asian nativity subgroup’s institutionalization rates were significantly different from US-born women in 1940.

**Figure 3:**
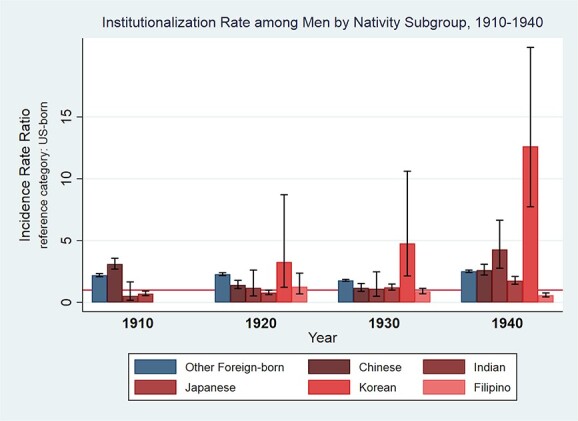
Institutionalization rates by nativity subgroup, men, 1910–1940.

Figure 3 displays corresponding results for men. The census did not report any Korean or Filipino men institutionalized in 1910. In 1910, Chinese men had a statistically higher institutionalization rate than all other nativity groups, and Japanese men had a lower rate than Chinese-, other foreign-born, and US-born men. In 1920, Japanese men had a lower institutionalization rate than other foreign- and Korean men. In 1930, Korean men had a higher institutionalization rate than Chinese-, Japanese-, Filipino-, other foreign-born, and US-born men, with no statistically significant differences among most other nativity groups. Chinese, Indian, Japanese, and Korean men had higher institutionalization rates than US-born men in 1940. Filipino men had a lower institutionalization rate than all other nativity subgroups, and Japanese men had a lower institutionalization rate than Chinese and Indian men in 1940.

### Descriptive Statistics: Sterilization Data

Table 3 displays demographic characteristics of individuals recommended for sterilization from 1920 to 1945 (*n* = 13,499) by nativity group with *P*-values for chi-square tests of independence across nativity groups. A greater proportion of US-born people were recommended for sterilization at young ages (29.62% younger than 20) when compared with Asian-born populations (6.01% younger than 20). A larger proportion of Asian-born people recommended for sterilization were men (70.38%) than among other foreign- (59.88%) or US-born (49.63%) people.

**Table 3:** Descriptive Statistics for Basic Demographic Information of Institutionalized Individuals Recommended for Sterilization in California by Nativity, 1920–1945. Totals Vary Due to Missing Information

	Asian-born	Other Foreign-born	US-born	
	*N* (%) or mean (SD)	*N* (%) or mean (SD)	*N* (%) or mean (SD)	*Χ* ^2^ *P*-value
Age				
<20	17 (6.01%)	170 (7.50%)	3,208 (29.62%)	—
20–29	102 (36.04%)	707 (31.20%)	4,064 (37.52%)	—
30–39	124 (43.82%)	1,001 (44.17%)	2,842 (26.24%)	—
40–49	38 (13.43%)	353 (15.58%)	658 (6.07%)	<0.0005
50–59	2 (0.71%)	34 (1.50%)	56 (0.52%)	—
60–69	—	1 (0.04%)	4 (0.04%)	—
Total	283	2266	10,832	—
Sex				
Female	85 (29.62%)	924 (40.12%)	5,495 (50.37%)	—
Male	202 (70.38%)	1,379 (59.88%)	5,414 (49.63%)	<0.0005
Total	287	2303	10,909	—
Institution				
Homes for the Feebleminded				
Sonoma	11 (3.83%)	140 (6.08%)	2,095 (19.21%)	—
Pacific Colony	1 (0.35%)	37 (1.61%)	1,207 (11.07%)	—
Total, Homes for the Feebleminded	12 (4.18%)	177 (7.69%)	3,302 (30.27%)	—
Hospitals for the Insane				
Patton	56 (19.51%)	606 (26.34%)	2,554 (23.41%)	—
Stockton	128 (44.60%)	746 (32.42%)	2,053 (18.82%)	—
Norwalk	11 (3.83%)	174 (7.56%)	770 (7.06%)	<0.0005
Agnews	9 (3.14%)	98 (4.26%)	655 (6.00%)	—
Mendocino	20 (6.97%)	145 (6.30%)	241 (2.21%)	—
Camarillo	2 (0.70%)	22 (0.96%)	138 (1.27%)	—
Napa	49 (17.07%)	333 (14.47%)	1,195 (10.96%)	—
Total, Hospitals for the Insane	275 (95.82%)	2,124 (92.31%)	7,606 (69.72%)	—
Total, all institutions	287	2301	10,908	—
Period				
1920–1924	35 (12.20%)	472 (20.48%)	657 (6.02%)	—
1925–1929	80 (27.87%)	593 (25.73%)	1,054 (9.66%)	—
1930–1934	54 (18.82%)	439 (19.05%)	1,778 (16.29%)	<0.0005
1935–1939	67 (23.34%)	494 (21.43%)	3,768 (34.52%)	—
1940–1945	51 (17.77%)	307 (13.32%)	3,657 (33.51%)	—
Total	287	2305	10,914	—

The institutions that sterilized the most Asian-born patients were Stockton (44.60% of Asian-born patients recommended for sterilization), Napa (17.07%), and Patton (19.51%). A greater proportion of Asian-born patients were sterilized at Stockton than were other foreign- (32.42%) or US-born (18.82%) persons. “Hospitals for the Insane” sterilized 95.82% of Asian- and 92.31% of other foreign-born patients recommended for sterilization. Although most US-born patients recommended for sterilization were also sterilized at “Hospitals for the Insane” (69.72%), the proportion of US-born patients sterilized at “Homes for the Feebleminded” (30.27%) was higher than for Asian- and other foreign-born patients.

The number of sterilizations among US-born patients increased from 1920 to 1939 and then decreased slightly from 1940 to 1945. The number of sterilizations among Asian- and other foreign-born patients did not increase or decrease steadily across multiple periods. Descriptives for patient histories, diagnosis, legal provision, and consent are presented in Table S1 of the supplement.

### Sterilization Rate Analysis

**Figure 4:**
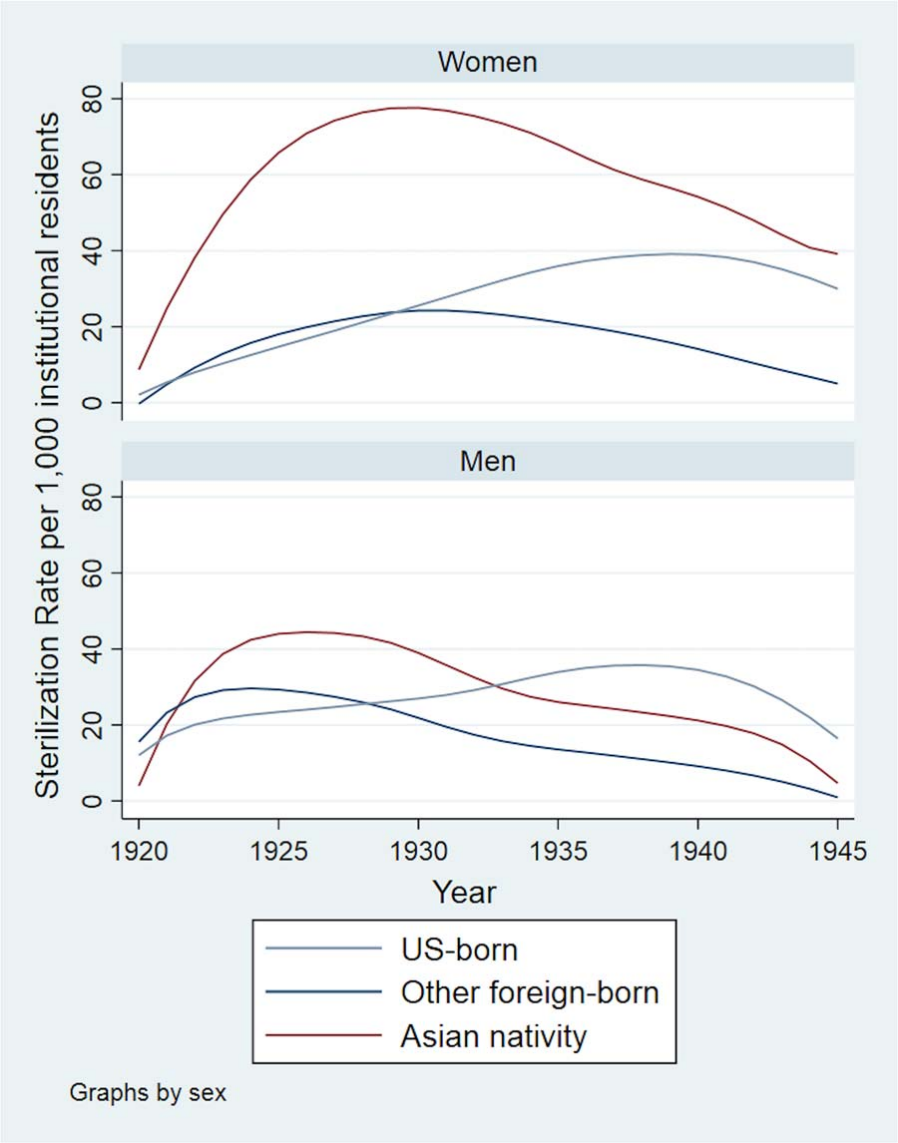
Locally Estimated Scatterplot Smoothing curves of sterilization rates among California’s institutionalized population stratified by gender and nativity group, 1920–1945.

Figure 4 presents LOESS curves of annual sterilization rates per 1,000 institution residents stratified by gender and nativity group. Asian-born women had the highest sterilization rate of the six groups across the study period. Asian-born men had higher sterilization rates than other foreign- and US-born men early in the study period before dropping below the sterilization rate for US-born men around 1933. The sterilization rate of Asian-born women was initially similar to that of Asian-born men but much higher for the remainder of the study period. Annual sterilization rates among Asian-born men peaked around 1925, and rates among Asian-born women peaked around 1930.

Table 4 presents the gender-stratified unadjusted Poisson regression model results, with US-born persons as the reference group. Asian-born women had significantly higher sterilization rates than US-born women [IRR = 2.00 (95% CI: 1.61, 2.48)] and other foreign-born women (*P*-value for difference in coefficients < 0.001). Asian-born men did not have significantly different rates of sterilization from US-born men [IRR = 0.95 (95% CI: 0.83, 1.10)] but did have significantly higher sterilization rates than other foreign-born men (*P*-value for difference in coefficients < 0.001). The online supplement presents sensitivity analysis LOESS curves in Figures S2 and S3 and regression results in Tables S2–S5.

**Table 4:** Poisson Regression Results, Stratified by Gender

	Women		Men	
	IRR (95% CI)	*p*	IRR (95% CI)	*p*
Nativity group				
US-born	(ref)	—	(ref)	—
Asian-born	2.00 (1.61, 2.48)^a^	<0.001	0.95 (0.83, 1.10)^b^	0.493
Other foreign-born	0.54 (0.51, 0.58)	<0.001	0.54 (0.51, 0.58)	<0.001
Constant	0.03 (0.03, 0.03)	<0.001	0.03 (0.03, 0.03))	<0.001

**Note:** Constant estimates incidence rate for reference group

*P*-values for difference between Asian- and other foreign-born coefficients:

^a^ *P* < 0.001

^b^ *P* < 0.001

Given the change in direction of relative sterilization rates for men by nativity group suggested by the LOESS curves, an indicator allowing different coefficients before and after 1933 was incorporated into another Poisson model for men (table 5). The inflection point model shows a change in the incidence rate ratio before and after 1933. Asian-born men had a higher sterilization rate than US-born men prior to 1933 [IRR 1.31 (95% CI 1.09, 1.59)] but a significantly lower sterilization rate [IRR 0.73 (0.59, 0.89)] after 1933. In both time periods, the coefficients for Asian-born men were significantly higher than the coefficients for other foreign-born men (*P* < 0.001 for before and after 1933).

**Table 5:** Sterilization Incidence Rate Ratios for Men, Before and After 1933

	IRR (95% CI)	*P*
Nativity group × time period		
US-born	(ref)	—
Asian-born (pre-1933)	1.31 (1.09, 1.59)^a^	0.005
Asian-born (1933 and later)	0.73 (0.59, 0.89)^b^	0.002
Other foreign-born (pre-1933)	0.86 (0.80, 0.93)	<0.001
Other foreign-born (1933 and later)	0.32 (0.29, 0.35)	<0.001
Constant	0.03 (0.03, 0.03)	<0.001

**Note:** Constant estimates incidence rate for reference group

*P*-values for difference between Asian- and other foreign-born coefficients:

^a^ *P* < 0.001

^b^ *P* < 0.001

## Discussion

Our study presents novel quantitative evidence of disproportionate eugenic sterilization of Asian immigrants in twentieth-century California and follows Asian-American studies scholars in centering the experiences of Asian immigrants in the history of the United States. We connect the racist and gendered implementation of Asian immigration restriction to the racism and sexism inherent in eugenic theories and practices. Though Asian immigrant women constituted a small proportion of California’s population during the height of its sterilization program, we confirmed our hypothesis of higher rates of sterilization of Asian-born women compared with US- and other foreign-born women from 1920 to 1945. Asian-born men only showed higher sterilization rates prior to 1933, in partial agreement with our initial hypothesis, for reasons requiring additional research to illuminate.

Contrary to our hypothesis of higher institutionalization rates of Asian immigrants, other foreign-born men and women consistently had the highest institutionalization rates across censuses while Asian-born men and women were generally institutionalized at similar rates to their US-born counterparts. However, disaggregating the Asian nativity group by national origin subgroup substantiated our expectation of heterogeneity in institutionalization rates by nativity subgroup.

### Institutionalization

Asian-born men and women were institutionalized at similar rates to their US-born counterparts, except in 1920 when Asian-born women had a lower institutionalization and 1910 and 1940 when Asian-born men had higher institutionalization rates. Immigrants were often blamed for high “insanity rates” and overcrowding in institutions in California (Fox 1978) and other states (Appleman 2018). Fox (1978) claims demographic factors, such as age, sex, and familial relationships, better explain disproportionate institutional immigrant populations than immigration status alone. Our results indicate race and national origin must also be considered in explaining disproportionate institutionalization rates of immigrants and broader manifestations of xenophobia and anti-immigrant discrimination (Sáenz and Douglas 2015). In future research, the disaggregation of the other foreign-born group could shed new light on the relationship between racism and immigration status in early institutional dynamics in California.

Indeed, further disaggregation of the Asian-born nativity group revealed in certain census years one national origin group drove the observed differences in aggregate institutionalization rates and could mask lower institutionalization rates of other groups. In 1910, Chinese-born men appeared to drive the higher institutionalization rate for Asian-born men compared with US-born men and mask the significantly lower rate for Japanese-born men. In 1940, Chinese-born men again contributed to the higher institutionalization rate of Asian-born men, now in conjunction with Indian-, Japanese-, and Korean-born men and masking the lower institutionalization rate for Filipino-born men relative to US-born men. Disaggregated rates for women revealed lower institutionalization rates for Japanese-born relative to US-born women in every census year except 1940. Our institutionalization rate findings for Japanese-born men and women parallel Molina’s (2006) observation of limited public health institutional intervention with the Japanese-American community in early-twentieth-century Los Angeles. More broadly, these findings support calls for the data disaggregation of Asian-Americans (Chan 2007; Gee et al. 2009; Lee 2010) in specific research contexts.

### Sterilization

Our finding of disproportionate sterilization of Asian immigrant women across the study period and men prior to 1933 strengthens the evidence documenting a racialized implementation of eugenic sterilization previously demonstrated for other racialized groups: Latino/a/x people in California and Black people in North Carolina (Novak et al. 2018; Price and Darity 2010; Price, Darity J, and Sharpe 2020). Unfortunately, no sterilization statistics by race or nativity exist from states with Asian populations comparable to California. Washington and Oregon had eugenic sterilization programs, albeit smaller ones. Eugenicists in Oregon were concerned about Asian populations, demonstrated by a special section entitled “Chinese and Japanese in Oregon” (Carlisle 1921, 22) in a statistical report investigating “basic causes of mental and physical disease or defect, and the relationship of such disorders to delinquency and dependency” (Carlisle 1921, v). In the context of our results for California, further study of eugenic sterilization in Western and Southwestern states is clearly needed to situate sterilization within broader Asian-American history.

Distinct gender differences persisted in sterilization rates of Asian-born individuals. Asian-born women were sterilized at a rate twice that of US-born women and had an even greater disparity with other foreign-born women. Furthermore, disproportionate sterilization of Asian-born women occurred despite lower rates of institutionalization of this group; the institutionalization rates of Asian-born women were statistically similar or lower than the rates of US- and other foreign-born women in every census year examined. Women can be an extremely small numerical presence and still play central roles in their communities as Mabalon (2013) astutely argues in her account of Stockton’s early-twentieth-century Filipino-American community. Scholars studying eugenics frequently center minority populations in examining the skewed implementation of sterilization practices. As research about Asian-American experiences with the eugenics movement and sterilization expands, our study serves as a reminder to not overlook Asian-American women who often exist as “a minority within a minority” (Mabalon 2013, 152).

In perhaps our most surprising finding, the sterilization rate of Asian-born men was not higher than the rate of other foreign- or US-born men until taking modification by the year of sterilization recommendation into account. This finding is somewhat consistent with other quantitative studies of eugenic sterilization in California (Novak et al. 2018) and other states (Reilly 1991) where sterilization rates for men peaked earlier than those for women. Our study similarly showed male sterilization rates peaking earlier in the study period than female rates but only for Asian- and other foreign-born men. Sterilization rates for US-born men peaked at a similar time to the rates for US-born women, meaning immigrants could drive the gender differential in the timing of peak sterilization rates in California. Thus, our study reaffirms the importance of incorporating gender into analyses of eugenic sterilization programs. Although Novak et al. (2018) accounted for gender in their analysis, two quantitative studies of North Carolina’s eugenics program (Price and Darity 2010; Price, Darity, and Sharpe 2020) could not because the sterilization counts by county used in the analyses lacked sex information. The gendered findings of this study suggest gender remains an underexplored aspect of eugenics in North Carolina and possibly other states.

The greater effect size in the regression models for Asian-born women than in those for Asian-born men strongly contradicts the subordinate male target hypothesis under which Asian-born men would be the primary targets of racism toward Asian immigrants as perceived sexual and reproductive threats to the white US-born population (Navarrete et al. 2010; Sidanius et al. 2018). Intersectionality Theory and the Gendered Racism Model (Crenshaw 2018; Espiritu 1997; Liu and Wong 2018) best explain our results, wherein Asian women and men experienced unique but related discrimination within California’s eugenic sterilization program. Institutionalization (Radford 1991), deportation (Stern 2016), and exploitative labor practices (Espiritu 1997; Price, Darity Jr., and Sharpe 2020; Shah 2011) as potential forms of eugenically motivated discrimination may have manifested more strongly for Asian-born men than for women, whereas similar eugenic motivations may have manifested as a greater disproportionality in sterilization rates for Asian-born women over a longer period. Our results indicating Asian-born men only had higher sterilization rates than the US-born men “in group” for part of the study period are perhaps less surprising in this context.

The specificity of the 1933 inflection point remains less easily explained and requires additional research to clarify. The relative sterilization rates clearly flipped for US- and Asian-born men in 1933 and led to our purely data-driven decision to use 1933 as the year in our inflection point models. We believe this shift is more than a demographic artifact of fluctuating labor needs and declining immigration of Asian male laborers due to exclusion laws because we account for changes in the institutionalized population with the inclusion of census denominators. Nevertheless, immigration restriction or momentous economic changes could have shifted how eugenicists and American society viewed Asian-born men as economic, social, and political “threats” to white Californians. For example, the Great Depression’s onset several years before and the Tydings–McDuffie act reclassifying Filipinos from US nationals to aliens ineligible for citizenship one year after the observed change in relative sterilization rates represents major transformations with the power to potentially change the application of eugenic sterilization to men of different groups. Although we can currently only speculate, the above two events in addition to other contextual factors such as population size, labor participation, political power, and community visibility constitute intriguing avenues of future research into historical eugenic sterilization.

### Sensitivity Analysis

While nativity had less missingness than race, the missingness may still have biased the results. In sensitivity analyses imputing US nativity for all missing nativity data, Asian-born men exhibited lower sterilization rates than their US-born counterparts. The corresponding version of the inflection point model resulted in statistically similar sterilization rates for Asian- and US-born men prior to 1933. However, imputing US nativity for all sterilization survivors missing nativity data still resulted in significantly higher sterilization rates for Asian-born relative to US-born women, albeit with a smaller effect size. The other set of sensitivity analyses extended the nativity distribution of sterilization survivors from Stockton State Hospital to all sterilization survivors missing nativity data. The Poisson regression models resulted in greater effect sizes in Asian-born women’s higher sterilization rates across the study period and Asian-born men’s sterilization rates before 1933.

Nativity information was missing more often for women than for men: 26.12% of women versus 22.09% of men recommended for sterilization. This differential missingness likely contributed to wider variation in upper and lower sensitivity estimates for women compared with men. Yet our finding of higher sterilization rates of Asian-born relative to US- and other foreign-born women held even after accounting for missing data. Thus, our sensitivity analysis results strengthen the evidence for our conclusion that Asian-born women were sterilized at disproportionately higher rates compared with other foreign- and US-born women under California’s eugenic sterilization program.

### Strengths

This study drew strength from its population-based design, ensuring adequate size for analysis. Despite small counts in both the census and sterilization recommendation datasets, LOESS analysis resulted in stable sterilization rates and regressions in acceptable model fit parameters. Using nativity instead of race allowed for less missing data, narrowed the population of interest and theoretical focus to immigrants, addressed some potential racial misclassification, and demonstrated the nuance of disaggregating subgroups comprising a racialized group. Another strength was examining both institutionalization and sterilization rates since California primarily sponsored the sterilization of institutional residents. In addition, this study identified differences in consent, diagnoses, and descriptive characteristics of Asian-born sterilization survivors. Finally, these findings can strengthen the foundation for continued quantitative and qualitative study of the United States’s history of eugenics and motivate new research questions.

### Limitations

Unfortunately, we could not adjust models for age and institution because small cell counts prevented reliable interpolation between census years when incorporating additional variables. Statistically significant demographic differences between nativity groups and the population recommended for sterilization versus the institutionalized population suggest age and institution influence sterilization patterns, confounding the results. Asian individuals in some nativity subgroups resided in geographic and ethnic clusters in California (Molina 2006; Ngai 2014; Shah 2001; Takaki 1998), and if incarceration in specific institutions depended upon residential location, institutional confounding could have attenuated or exaggerated the effect.

Additionally, large confidence intervals for institutionalization rates by nativity subgroup limit meaningful conclusions about nuances of national origin and institutionalization. Korean institutionalization rates appeared especially high compared with other nativity subgroups, though small numbers and possible misclassification due to the Japanese occupation of Korea in 1910–1945 mean these numbers should be interpreted cautiously. Moreover, we could not examine differential sterilization rates among nativity subgroups due to small cell sizes preventing a reliable interpolation of census denominators. Future research could explore the nuances of Asian subgroup differences in institutionalization and sterilization rates with more complex methods, such as surname analysis.

Our assumption that a sterilization request form indicates a sterilization operation occurred may have misclassified some individuals who should have been part of the at-risk institutionalized population in the denominator rather than the population sterilized in the numerator. If misclassification was non-differential by nativity, it would bias the effect toward the null. The Spanish surname population, coded as other foreign- or US-born except some Filipino individuals with Spanish-influenced surnames, may have dampened the effect as well since they were another racialized group with sterilization rates disproportionate to their population share. Finally, using nativity to measure membership in a racialized Asian immigrant group may bias results toward the null due to “misclassifying” second-generation Asian-Americans. Due to perceptions of Asian-Americans as “perpetual foreigners,” they may have experienced similar discrimination in institutionalization and sterilization processes despite not being immigrants themselves.

## Conclusion

The historical rhetoric portraying Asians as perpetual foreigners and threats to public health bears unfortunate resemblance to increased racist and xenophobic rhetoric directed toward Asian-Americans and Asian immigrants during the COVID-19 pandemic. Asian-Americans have grown faster than any other major US racial group in the last two decades (López, Ruiz, and Patten 2017), increasing the importance of understanding how historical patterns of racial discrimination toward Asian-Americans may be enacted in contemporary public health discourse. Public health and medical science historically embraced ideologies of biological differences among races to justify discrimination and structural racism, erroneous ideas that still manifest in more subtle ways in genetics and other fields (McCabe and McCabe 2011; Winfield 2012). Quantitative and qualitative studies of historical eugenics can produce richer context in addressing the health impact of modern discrimination against Asians in the United States.

More recent reports of forced or coercive sterilization, such as California’s prison system sterilizing an estimated 148 incarcerated women without consent (Chappell 2013) and sterilization abuse reports from a US Immigration and Customs Enforcement detention facility (Project South et al. 2020), exemplify the ongoing need to confront eugenic legacies to achieve reproductive justice. California recently passed legislation to compensate sterilization survivors, which include those sterilized under its eugenics law and in prisons after 1979. A 2016 study estimated up to 831 survivors of coercive eugenic sterilizations in California may still be alive, and their experiences and the racial injustices wrought by these institutions deserve acknowledgment (Stern et al. 2017).

## Supplementary Material

sf-jul-21-354-File006_soad060

sf-jul-21-354-File007_soad060

sf-jul-21-354-File008_soad060

sf-jul-21-354-File009_soad060

## Data Availability

The data analyzed in this study is subject to the following licenses/restrictions: *Census data*—Researchers can access restricted complete count data (including names and string variables) for US Censuses 1870–1940 through a research agreement with IPUMS USA. Requests to access these datasets should be directed to ipums@umn.edu. *Sterilization data*—The data underlying this article cannot be shared publicly because they contain identifiable information associated with traumatic historical events. The data will be shared on reasonable request to the corresponding author.

## About the Authors

**Marie Kaniecki**, MPH, is a research data analyst at the Institute for Society and Genetics at the University of California, Los Angeles. She is interested in social determinants of health and the intersection of health and human rights. Her research focuses on using archival records in quantitative and mixed-methods analyses rooted in the principles of reproductive justice, disability justice, and racial justice.

**Nicole L. Novak**, PhD, MSc, is a research assistant professor at the University of Iowa College of Public Health. She uses epidemiologic and community-engaged research methods to examine historical, structural, and policy influences on health. She recently coauthored the article “Victims of eugenic sterilisation in Utah: cohort demographics and estimate of living survivors” in *The Lancet Regional Health—Americas* (2023).

**Sarah Gao**, MPH, currently works as a research assistant at the Harvard Center of Population and Development Studies on the Health and Aging in Africa: a Longitudinal Study of an INDEPTH community in South Africa. Her current research interests include cognitive aging and social determinants of health over the life course in low- and middle-income countries.

**Natalie Lira** is an associate professor in the Departments of Latina/Latino Studies and Gender and Women’s Studies at the University of Illinois at Urbana Champaign. Her research interests include the politics of reproduction, histories of medicine, and the ways that struggles for racial and reproductive justice intersect. She is the author of *Laboratory of Deficiency: Sterilization and Confinement in California, 1910–1950s* (University of California Press, 2021).

**ToniAnn Treviño**, PhD, is an assistant professor of History at Texas Lutheran University. Her manuscript in development examines how the war on drugs impacted ethnic Mexicans in the postwar Texas-Mexico borderlands. Her article, “Documenting Deviance: The Texas Department of Public Safety and Drug Raids in Mexican Neighborhoods, 1955-1960,” will appear in the July 2025 edition of the *Southwestern Historical Quarterly*.

**Kate O’Connor**, MPH, MA, is a PhD Candidate in American Culture at the University of Michigan. Her research focuses on the intersection of disability, race, gender, and sexuality in the Great Lakes region from the mid-nineteenth century to present. Her current project is a dissertation that focuses broadly on the history of eugenics in Michigan, with a focus on institutionalized children, adolescents, and young adults.

**Alexandra Minna Stern**, PhD, is the Dean of Humanities and Professor in the Department of English, History Department, and Institute for Society and Genetics at the University of California, Los Angeles. She is the author of the award-winning *Eugenic Nation: Faults and Frontiers of Better Breeding in Modern America* (2nd. ed, 2015) and *Proud Boys and the White Ethnostate: How the Alt-Right is Warping the American Imagination* (2019).

## Notes

The authors would like to thank Siobán Harlow, PhD, and Sharon Kardia, PhD, for contributing their quantitative expertise to the Sterilization and Social Justice Lab (SSJL). We would also like to thank the entire SSJL team for their support and feedback, especially Juan Gudino, MPH, and Elyse Thulin, MS. Additionally, we extend gratitude to Belinda Needham, PhD, for her mentorship of the lead author in the beginning stages of this analysis.

This research was financially supported by the National Human Genome Research Institute (R01 HG010567-01 and R21-HG009205-01) and University of Michigan MCubed (Project #526). We are grateful to IPUMS USA and the University of Michigan Institute for Social Research for facilitating access to historical census microdata and providing technical assistance.
