# Supplementary material for: Racialization and Reproduction: Asian Immigrants and California’s Twentieth-Century Eugenic Sterilization Program
Source: Soc Forces. 2023 Apr 29;102(2):706–29. doi: 10.1093/sf/soad060 (PMC10569381; doi:10.1093/sf/soad060)
Supplement: sf-jul-21-354-File006_soad060 [file sf-jul-21-354-file006_soad060.pdf]

Racialization and Reproduction:
Asian Immigrants and California's 20th Century Eugenic Sterilization Program

Supplement

Statistical Model

The Poisson model used for estimating and comparing sterilization rates using denominators from Census data will be:

$$\ln(\mu_{gj})=\ln(n_{gj})+\beta_0+\beta_g x_g$$

where *μ* is the count of sterilizations taking place in nativity group *g* and year *j*, and *n* is the total number of institutionalized individuals in the same nativity group and year. The covariate x_g is the categorical variable indicating nativity group *g.* Separate models will be estimated for men and for women.

Detailed Descriptive Statistics: Sterilization Data

The sterilization dataset coded patient histories summarized in recommendation forms as binary indicators (mentions of sexual delinquency, alcoholism/drug use, immigration, and disparaging reference(s) to patient's race). The breadth of diagnoses given in the forms necessitated categorization by the authors based on historical context: psychiatric or mental health diagnoses; cognitive, developmental, or physical disability; social or behavioral diagnoses; combinations of those three categories; and other. Psychiatric or mental health diagnoses, such as "manic depressive" and "psychosis", encompassed diagnoses that would roughly fall under the category of mental illness. Cognitive, developmental, or physical disabilities included various forms of "mental retardation" and blindness, epilepsy, and other physical disabilities. Social or behavioral diagnoses included alcoholism, substance abuse, and sexually transmitted infections as well as diagnoses not considered to be "medical" conditions today, such as sexual deviancy and criminal delinquency. Many individuals were given multiple diagnoses that fell into more than one category.

While diagnosis provided the so-called medical justification for sterilization, California law provided four specific legal provisions for sterilization: mental disease which may have been inherited and is likely to be transmitted to descendants, feeblemindedness in any of its various grades, perversion or marked departures from normal mentality, and/or disease of a syphilitic nature. Epilepsy was also given as the legal provision on some forms. Most people recommended for sterilization had both a diagnosis and the specific legal provision for the sterilization request listed on their forms.

The final descriptive statistics examined "consent" dynamics by nativity group. Consent was recorded as yes or no and typically referred to consent by family, legal guardian, or government or health officials rather than the patient themselves. Even cases accompanied by signed consent forms would be considered coercive under contemporary standards of informed consent. Some forms explained lack of consent, and the reasons were categorized as: no consenter available; no response to request; refused to consent; multiple reasons; still seeking consent; and other.

Table S1. Descriptive statistics for individuals recommended for sterilization in California by nativity, 1920-1945. Totals vary due to missing information. Chi-squared p-values were corrected for multiple testing using the Holm-Bonferroni method.

	Asian Born	Other Foreign Born	US Born	
	N (%) or Mean (SD)	N (%) or Mean (SD)	N (%) or Mean (SD)	X^2 p-value
**Marital Status**				
Single	96 (48.98%)	696 (42.21%)	5976 (61.79%)	<0.0005
Married	86 (43.88%)	797 (48.33%)	2757 (28.51%)	
Divorced	7 (3.57%)	118 (7.16%)	808 (8.35%)	
Widowed	6 (3.06%)	32 (1.94%)	112 (1.16%)	
Other	1 (0.51%)	6 (0.36%)	18 (0.19%)	
Total	196	1649	9671	
**Number of Children**				
	1.31 (2.07)	1.42 (2.13)	0.72 (1.43)	<0.0005
**Religion**				
Catholic	56 (43.08%)	717 (63.34%)	2,695 (32.21%)	<0.0005
Protestant	20 (15.38%)	292 (25.80%)	4,842 (57.87%)	
Other/Unspecified Christian	6 (4.62%)	37 (3.27%)	295 (3.53%)	
Hindu	1 (0.77%)	0 (0.00%)	0 (0.00%)	
Muslim	0 (0.00%)	1 (0.09%)	5 (0.06%)	
Jewish	2 (1.54%)	62 (5.48%)	143 (1.71%)	
“Japanese Religion”	4 (3.08%)	0 (0.00%)	2 (0.02%)	
“Chinese Religion”	3 (2.31%)	0 (0.00%)	0 (0.00%)	
Confucianism	4 (3.08%)	0 (0.00%)	1 (0.01%)	
Buddhist	23 (17.69%)	0 (0.00%)	29 (0.35%)	
Other	0 (0.00%)	1 (0.09%)	15 (0.18%)	
None	11 (8.46%)	22 (1.94%)	341 (4.08%)	
Total	130	1,132	8,368	
**Patient History**				
Sexual delinquency	12 (4.18%)	123 (5.34%)	1114 (10.21%)	<0.0005
Alcoholism/drug use	8 (2.79%)	85 (3.69%)	253 (2.32%)	0.001
Disparaging reference to race	8 (2.79%)	27 (1.17%)	110 (1.01%)	0.018
Immigrant	179 (62.37%)	1394 (60.48%)	16 (0.15%)	<0.0005

**Diagnosis**				
Psychiatric/Mental	219 (77.66%)	1464 (66.24%)	5237 (49.35%)	<0.0005
Cognitive/Developmental or Physical Disability	15 (5.32%)	234 (10.59%)	3435 (32.37%)	
Social/Behavioral	1 (0.35%)	31 (1.40%)	112 (1.06%)	
Psychiatric/Mental Health and Cognitive/Developmental or Physical Disability	8 (2.84%)	149 (6.74%)	793 (7.47%)	
Psychiatric/Mental Health and Social/Behavioral	26 (9.22%)	168 (7.60%)	474 (4.47%)	
Cognitive/Developmental or Physical Disability and Social/Behavioral	3 (1.06%)	27 (1.22%)	203 (1.91%)	
All Three Categories	0 (0.00%)	11 (0.50%)	36 (0.34%)	
Other	10 (3.55%)	126 (5.70%)	322 (3.03%)	
Total	282	2210	10612	
**Legal Provision**				
Mental Disease	255 (92.39%)	1940 (89.94%)	6801 (64.88%)	<0.0005
Feeblemindedness	5 (1.81%)	121 (5.61%)	2694 (25.70%)	
Perversion/Departure from normal mentality	0 (0.00%)	10 (0.46%)	149 (1.42%)	
Syphilis	8 (2.90%)	13 (0.60%)	109 (1.04%)	
Epilepsy	0 (0.00%)	5 (0.23%)	36 (0.34%)	
Other	1 (0.36%)	5 (0.23%)	34 (0.32%)	
Multiple	7 (2.54%)	63 (2.92%)	660 (6.30%)	
Total	276	2157	10483	
**Consent**				
No signed consent form available	164 (57.34%)	898 (38.96%)	1834 (16.81%)	<0.0005
Where consent is missing, reason for lack of consent				
No consenter available	101 (44.69%)	307 (17.21%)	617 (6.07%)	<0.0005
No response to request	2 (0.88%)	32 (1.79%)	219 (2.15%)	
Refused to consent	1 (0.44%)	18 (1.01%)	169 (1.66%)	
Multiple reasons	0 (0.00%)	4 (0.22%)	43 (0.42%)	
Still seeking consent	0 (0.00%)	4 (0.22%)	16 (0.16%)	
Other	0 (0.00%)	12 (0.67%)	22 (0.22%)	
Total	104	377	1086	

Sensitivity Analysis

A large number of people in the sterilization dataset (n=4,282) were missing information on nativity. The sensitivity analysis considers the potential ways this missingness could affect the study findings. We present two sets of models, each with a different assumption about the nativity of those with missing data.

The first, “lower-bound” model, assumes that all individuals recommended for sterilization with missing nativity information were US-born. The second, “upper bound” sensitivity analysis assumes that the group of people missing nativity data have a similar nativity distribution to the population of patients at Stockton State Hospital. The proportion Asian-born, by year and sex (average=4.34% Asian-born, minimum=0% Asian-born in 1921, and maximum=10.2% Asian-born in 1928) were imputed to those missing nativity at all institutions. Stockton was chosen to represent a plausible upper bound for proportions of those with Asian nativity because 44.59% of all Asian-born individuals recommended for sterilization were institutionalized at Stockton and only 3.44% of those recommended for sterilization with missing nativity were institutionalized at Stockton.

Lower Bound Estimate

Table S2. Poisson Regression results, stratified by gender, lower bound sensitivity analysis

	**Women**		**Men**	
	IRR (95% CI)	p	IRR (95% CI)	p
Nativity group				
US-born	(ref)		(ref)	
Asian-born	1.41 (1.14, 1.74)[a]	0.002	0.70 (0.61, 0.80)[b]	<0.001
Other foreign-born	0.38 (0.36, 0.41)	<0.001	0.42 (0.38, 0.42)	<0.001
Constant	0.04 (0.04, 0.05)	<0.001	0.04 (0.04, 0.04))	<0.001

Note: constant estimates incidence rate for reference group
[a,b]p-values for difference between Asian-born and other-foreign born coefficients: a: *p<0.001*; b: *p<0.001*

Table S3. Sterilization Incidence Rate Ratios for men, before and after 1933, lower bound sensitivity analysis

	IRR (95% CI)	p
Nativity group*time period		
US-born	(ref)	
Asian-born (pre-1933)	0.96 (0.79, 1.16)[a]	0.689
Asian-Born (post-1933)	0.53 (0.43, 0.65)[b]	<0.001
Other-Foreign Born (pre-1933)	0.63 (0.59, 0.68)	<0.001
Other-Foreign Born (post-1933)	0.23 (0.21, 0.26)	<0.001
Constant	0.04 (0.04, 0.04)	<0.001

Note: constant estimates incidence rate for reference group
[a,b]p-values for difference between Asian-born and other-foreign born coefficients: a: *p=0.001*; b: *p<0.001*

Upper Bound Estimate

Table S4. Poisson Regression results, stratified by gender, upper bound sensitivity analysis

	**Women**		**Men**	
	IRR (95% CI)	p	IRR (95% CI)	p
Nativity group				
US-born	(ref)		(ref)	
Asian-born	3.64 (3.18, 4.17)[a]	<0.001	1.03 (0.92, 1.16)[a]	0.605
Other foreign-born	0.39 (0.37, 0.42)	<0.001	0.41 (0.38, 0.43)	<0.001
Constant	0.04 (0.04, 0.04)		0.04 (0.04, 0.04)	<0.001

Note: constant estimates incidence rate for reference group
[a,b]p-values for difference between Asian-born and other-foreign born coefficients: a: $p<0.001$; b: $p<0.001$

Table S5. Sterilization Incidence Rate Ratios for men, before and after 1933, upper bound sensitivity analysis

	IRR (95% CI)	p
Nativity group*time period		
US-born	(ref)	
Asian-born (pre-1933)	1.63 (1.41, 1.89)[a]	<0.001
Asian-Born (post-1933)	0.66 (0.55, 0.79)[b]	<0.001
Other-Foreign Born (pre-1933)	0.64 (0.60, 0.69)	<0.001
Other-Foreign Born (post-1933)	0.24 (0.22, 0.26)	<0.001
Constant		

Note: constant estimates incidence rate for reference group
[a,b]p-values for difference between Asian-born and other-foreign born coefficients: a: $p<0.001$; b: $p<0.001$
