# Supplementary figures and images for: Racialization and Reproduction: Asian Immigrants and California’s Twentieth-Century Eugenic Sterilization Program
Source: Soc Forces. 2023 Apr 29;102(2):706–29. doi: 10.1093/sf/soad060 (PMC10569381; doi:10.1093/sf/soad060)

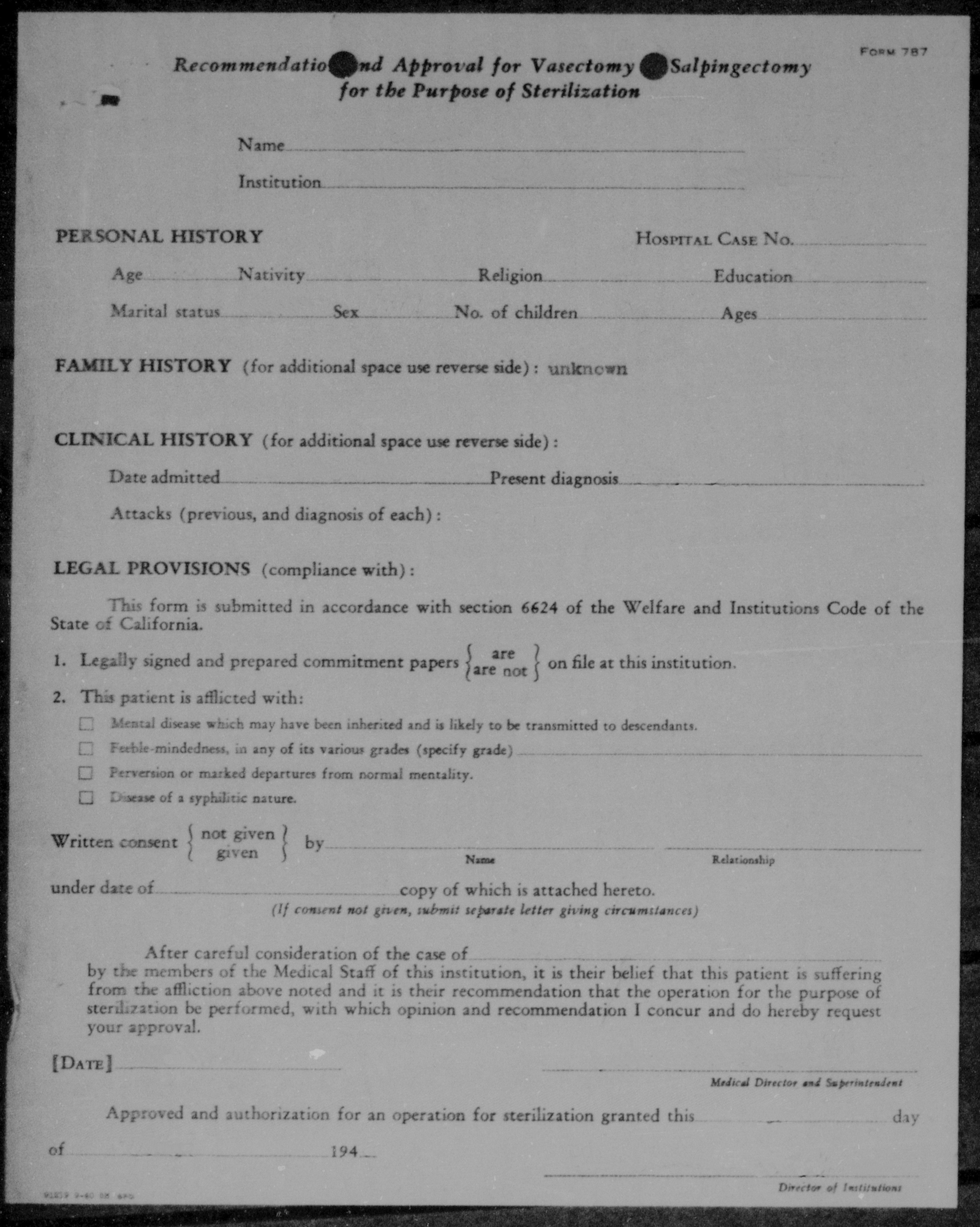

Supplement: sf-jul-21-354-File007_soad060 [file sf-jul-21-354-file007_soad060.jpeg]

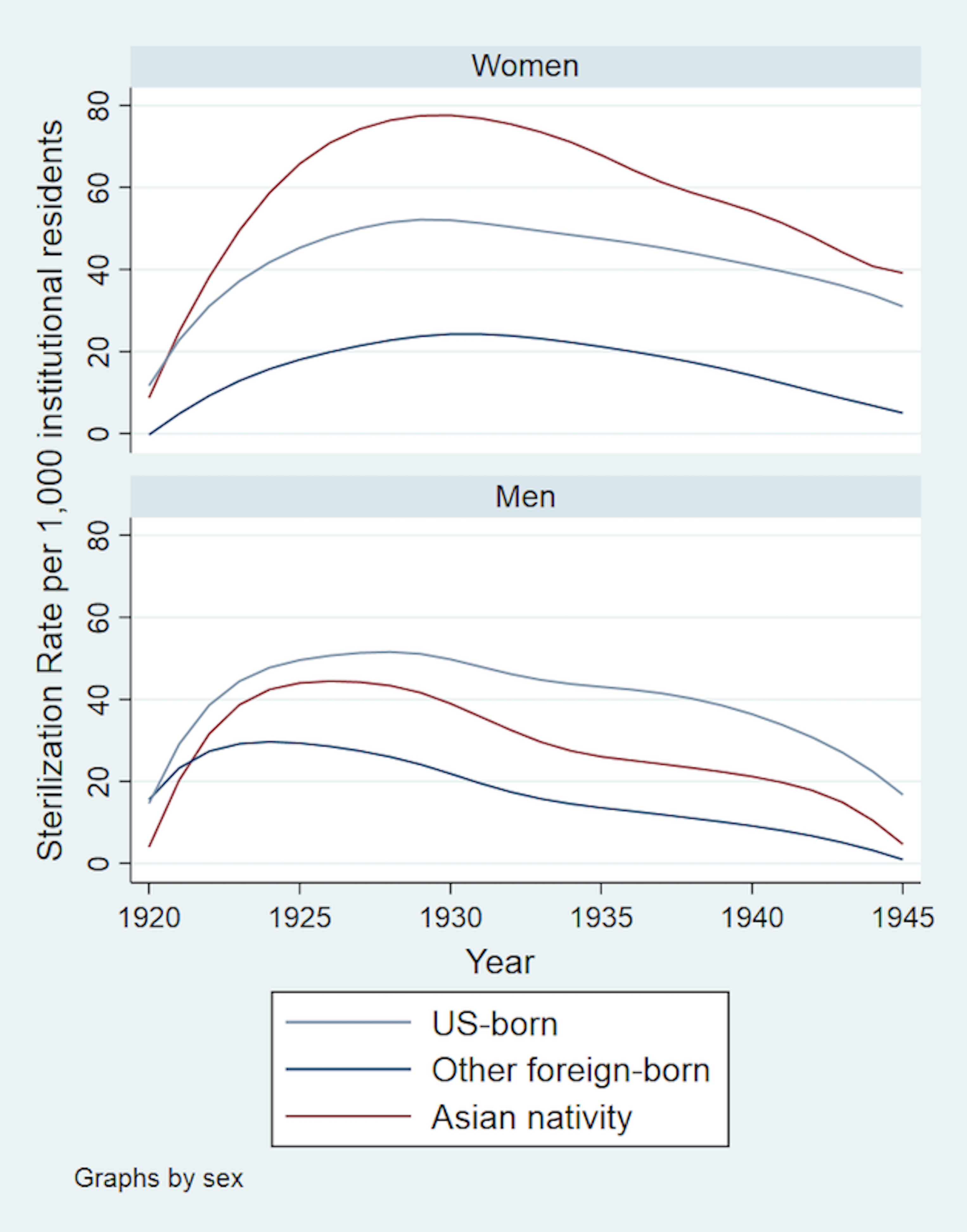

Supplement: sf-jul-21-354-File008_soad060 [file sf-jul-21-354-file008_soad060.jpeg]

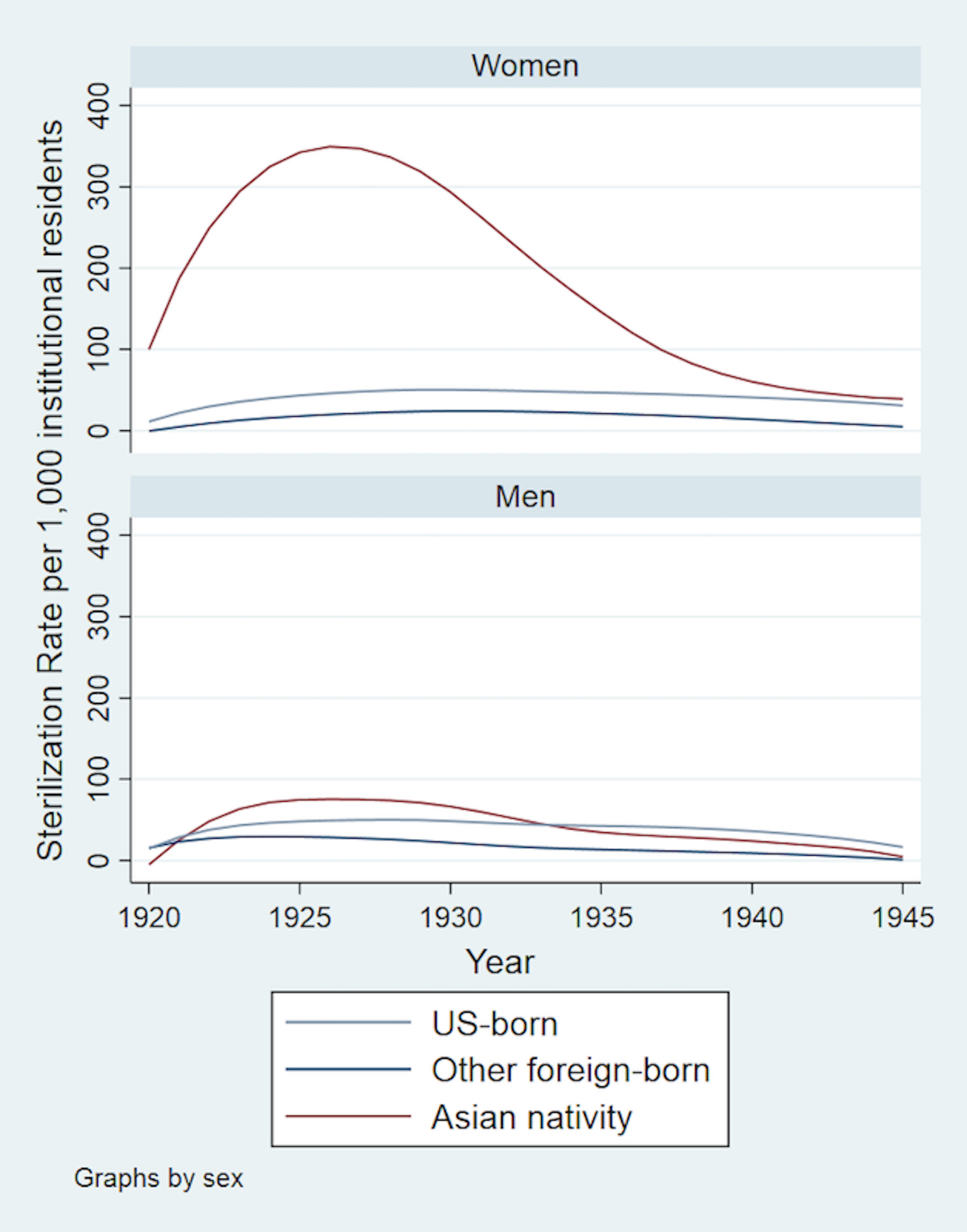

Supplement: sf-jul-21-354-File009_soad060 [file sf-jul-21-354-file009_soad060.jpeg]
